# DL-3-n-butylphthalide improved physical and learning and memory performance of rodents exposed to acute and chronic hypobaric hypoxia

**DOI:** 10.1186/s40779-021-00314-7

**Published:** 2021-03-25

**Authors:** Gang Xu, Yi-Kun Shi, Bin-Da Sun, Lu Liu, Guo-Ji E., Shu He, Jian-Yang Zhang, Bao Liu, Qiu Hu, Jian Chen, Yu-Qi Gao, Er-Long Zhang

**Affiliations:** 1 Institute of Medicine and Equipment for High Altitude Region, College of High Altitude Military Medicine, Army Medical University, Number 30, Gaotanyan Street, District of Shapingba, Chongqing, 400038 China; 2 Key Laboratory of Extreme Environmental Medicine, Ministry of Education of China, Chongqing, China; 3 Key Laboratory of High Altitude Medicine, People’s Liberation Army, Chongqing, China

**Keywords:** DL-3-n-butylphthalide, Hypobaric hypoxia, Physical function, Learning and memory function, Oxidative stress, Energy metabolism

## Abstract

**Background:**

Studies have revealed the protective effect of DL-3-n-butylphthalide (NBP) against diseases associated with ischemic hypoxia. However, the role of NBP in animals with hypobaric hypoxia has not been elucidated. This study investigated the effects of NBP on rodents with acute and chronic hypobaric hypoxia.

**Methods:**

Sprague-Dwaley rats and Kunming mice administered with NBP (0, 60, 120, and 240 mg/kg for rats and 0, 90, 180, and 360 mg/kg for mice) were placed in a hypobaric hypoxia chamber at 10,000 m and the survival percentages at 30 min were determined. Then, the time and distance to exhaustion of drug-treated rodents were evaluated during treadmill running and motor-driven wheel-track treadmill experiments, conducted at 5800 m for 3 days or 20 days, to evaluate changes in physical functions. The frequency of active escapes and duration of active escapes were also determined for rats in a shuttle-box experiment, conducted at 5800 m for 6 days or 27 days, to evaluate changes in learning and memory function. ATP levels were measured in the gastrocnemius muscle and malonaldehyde (MDA), superoxide dismutase (SOD), hydrogen peroxide (H_2_O_2_), glutathione peroxidase (GSH-Px), and lactate were detected in sera of rats, and routine blood tests were also performed.

**Results:**

Survival analysis at 10,000 m indicated NBP could improve hypoxia tolerance ability. The time and distance to exhaustion for mice (NBP, 90 mg/kg) and time to exhaustion for rats (NBP, 120 and 240 mg/kg) significantly increased under conditions of acute hypoxia compared with control group. NBP treatment also significantly increased the time to exhaustion for rats when exposed to chronic hypoxia. Moreover, 240 mg/kg NBP significantly increased the frequency of active escapes under conditions of acute hypoxia. Furthermore, the levels of MDA and H_2_O_2_ decreased but those of SOD and GSH-Px in the sera of rats increased under conditions of acute and chronic hypoxia. Additionally, ATP levels in the gastrocnemius muscle significantly increased, while lactate levels in sera significantly decreased.

**Conclusion:**

NBP improved physical and learning and memory functions in rodents exposed to acute or chronic hypobaric hypoxia by increasing their anti-oxidative capacity and energy supply.

**Supplementary Information:**

The online version contains supplementary material available at 10.1186/s40779-021-00314-7.

## Background

Exposure to a high altitude environment may lead to substantial decreases in work and cognitive performances [1, 2]. At an altitude of 4500 m, the maximum working capacity was found to be reduced to 50% of that observed at low altitude [2]. People exposed to hypobaric hypoxic conditions display significant alterations in cognitive processes, including attention span, short-term memory, decision making, simple and complex reaction times, and mood [1]. The changes that occur in physical and cognitive functions in response to hypobaric hypoxia may greatly affect work and normal life. Therefore, the development of drugs and methods to alleviate physical and cognitive impairment is imperative for those located at high altitudes for extended periods.

High altitude acclimatization is a physiological process that comprises a number of responses by different systems in the body, which take place upon exposure to plateau hypoxia. Many studies have revealed that the changes involved in acclimatization occur in various systems and with varying time courses. Many people can maintain the homeostasis needed for normal bodily function through acclimatization at a low oxygen partial pressure [3, 4]. However, acute and chronic high altitude illness will occur in people with poor acclimatization and can significantly damage diverse functionalities [5, 6]. Therefore, studying the molecular mechanisms of high altitude diseases may identify pathways that will enhance human capabilities at high altitude. Recent studies found that disorders of the inflammation response [7] and metabolism [8] as well as oxidative stress [9] were closely associated with high altitude illnesses. Agents that modulate these processes have the potential to improve human activities at high altitude.

DL-3-n-butylphthalide (NBP), a racemic mixture of an optical isomer extracted from the seeds of *Apium graveolens* Linn. (Fig. S1), is widely used to treat patients with ischemic stroke [10]. NBP is thought to inhibit inflammation and oxidative as well as endoplasmic reticulum stress and promote angiogenesis in animals and humans with cerebral ischemia [11]. NBP was recently shown to exert neuroprotective effects by alleviating vascular cognitive impairment [12] and promoting neuroplasticity and motor recovery after cerebral ischemia [13] and chronic intermittent hypoxia-hypercapnia [14]. Whether NBP exerts beneficial effects under other hypoxic conditions such as hypobaric hypoxia is, however, unclear.

In this work, we investigated the effects of NBP on the physical and cognitive abilities of rodents under hypobaric hypoxia conditions (equivalent to 5800 m). The effects of NBP on rodents’ behavior were evaluated through exhaustive exercise and shuttle-box experiments. We evaluated the potential mechanism of NBP by collecting muscle and blood samples from treated rodents and analyzing the levels of ATP, malonaldehyde (MDA), superoxide dismutase (SOD), hydrogen peroxide (H_2_O_2_), lactate, and glutathione peroxidase (GSH-Px) as well as by performing routine blood tests.

## Methods

### Experimental rodents and NBP administration

Male pathogen-free Sprague-Dawley (SD) rats (6 to 8 weeks old, weighing 180–220 g) and male pathogen-free Kunming mice (6 weeks old, weighing 18–20 g) were used in this study. Rats and mice were obtained from the Laboratory Animal Center of Army Medical University, and the animal study protocol was approved by the Animal Care and Use Committee Guidelines of the Army Medical University. NBP (purity, 99.6%) was obtained from Shijiazhuang Pharma Group NBP Pharmaceutical Co., Ltd. (Shijiazhuang, Hebei, China). A total of 200 rats and 224 mice were used for seven separate experiments in our study. Rats or mice for each experiment were randomly divided into four groups respectively: control group (administered an equivalent volume of corn oil), NBP low dose-treated group (60 mg/kg for rats and 90 mg/kg for mice), NBP intermediate dose-treated group (120 mg/kg for rats and 180 mg/kg for mice), and NBP high dose-treated group (240 mg/kg for rats and 360 mg/kg for mice). The detailed plan for each experiment was described as followed. NBP was intragastrically administered once or twice every day. Rodents had free access to food and water.

### Acute hypoxia survival experiment at 10,000 m in a hypobaric hypoxia chamber

**Fig. 1:**
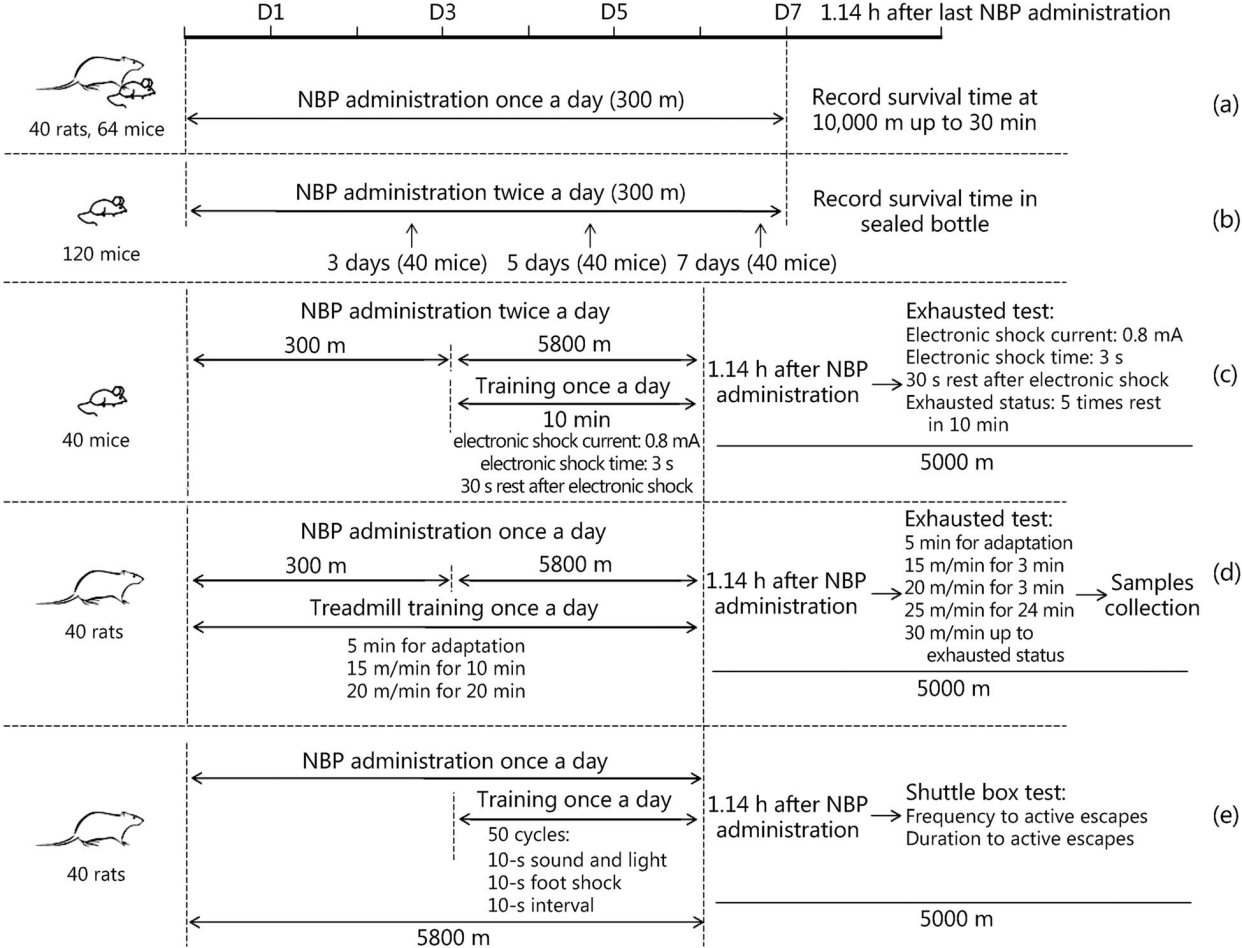
Diagrams depicting hypoxia tolerance experiments and experiments conducted under conditions of acute hypoxia. (**a**) Acute hypoxia survival experiment in a 10,000 m hypobaric hypoxia chamber; (**b**) standard hypoxia tolerance experiment for mice; (**c**) motor-driven wheel-track treadmill fatigue experiments for acute hypoxic mice; (**d**) treadmill running experiment for acute hypoxic rats; (**e**) shuttle-box experiment for acute hypoxic rats

The purpose of this experiment was to evaluate the effect of NBP on the hypoxia tolerance of rats and mice exposed to acute hypobaric hypoxia. Forty rats were randomly divided to four experimental groups with 10 rats in each group. Sixty four mice were randomly divided to four experimental groups with 16 mice in each group. NBP was given to SD rats and Kunming mice by intragastric administration at 1 ml/100 g body weight for 7 days at 300 m altitude (Chongqing altitude). At 1.14 h after the last administration, the rodents were placed in the hypobaric hypoxia chamber. The high altitude of the chamber ascended to 10,000 m at a rate of approximately 1000 m/min. The survival of rodents was observed and recorded [15] and each experiment was stopped after 30 min at 10,000 m (Fig. 1a).

### Standard hypoxia tolerance experiment of mice

This experiment was used to evaluate the effect of NBP on the hypoxia tolerance of mice under conditions of normobaric hypoxia. One hundred and twenty Kunming mice were separately used for experiments with NBP intragastric administration for 3 days, 5 days or 7 days, and forty mice were used for each time point. Forty mice of each time were randomly divided into four experimental groups with 10 mice in each group. At 1.14 h after the last administration, each mouse was put into a 125-ml bottle and 5 g of soda lime were added, which was used to absorb the carbon dioxide and water vapor produced by breathing. Then, the lid was tightly sealed until the mouse’s breathing movements stopped. The survival time (ST, min), i.e., the time from when the mouse was sealed in the bottle to its death, was recorded (Fig. 1b) [15], and standard hypoxia tolerance time (STT, min/ml) was calculated according to the following formula [16]:

$$ \mathrm{STT}=\mathrm{ST}/\left(V-\mathrm{BW}/0.94\right) $$

where V is the bottle volume (ml) and BW is the body weight of the mouse (g).

### Motor-driven wheel-track treadmill experiments for acute hypoxic mice

This experiment was used to evaluate the effect of NBP on the physical ability of mice under conditions of acute hypobaric hypoxia. Forty mice were randomly divided to four experimental groups with 10 mice in each group. Kunming mice were intragastrically treated with NBP twice per day for 6 days. Mice were raised at 300 m altitude for 3 days. On day 4, mice were placed at 5800 m altitude and exercised on a motor-driven wheel-track treadmill (YLS-10B, Shandong Academy of Medical Sciences, Jinan, China) for 3 days under the following conditions: 10 min/d, electronic shock current: 0.8 mA, electronic shock time: 3 s, 30 s rest after electronic shock, and five or more times rest in 10 min as exhausted standard [17]. On day 7, mice were performed exhaustion test on the motor-driven wheel-track treadmill 1.14 h after NBP administration at 5000 m altitude and the distance to exhaustion was recorded (Fig. 1c).

### Treadmill running experiment for acute hypoxic rats

This experiment was used to evaluate the effect of NBP on the physical ability of rats to run on a treadmill under conditions of acute hypobaric hypoxia. Forty rats were randomly divided to four experimental groups with 10 rats in each group. NBP was intragastrically administered to SD rats for 3 days at an altitude of 300 m. Rats were then subjected to treadmill exercise every day as follows: 5 min for adaptation, followed by 15 m/min for 10 min and 20 m/min for 20 min. On day 4, rats were placed in a 5800 m hypobaric hypoxia chamber, administered NBP and subjected to the above exercise regimen for 3 days. On day 7, an exhaustion test was conducted by extending the treadmill running experiment after NBP administration at 5000 m altitude. The experimental plan included 5 min for adaptation, followed by 15 m/min for 3 min, 20 m/min for 3 min, 25 m/min for 24 min, and then 30 m/min up to exhausted status [18]. The total running time was used to evaluate physical ability under acute hypoxia exposure. Rats were anaesthetized and their arterial blood was collected for analysis of MDA, SOD, H_2_O_2_, lactate, and GSH-Px levels and routine blood tests were also performed. In addition, the gastrocnemius muscle tissues were excised and used for ATP detection. MDA, SOD, H_2_O_2_, lactate, and GSH-Px levels were detected using Nanjing Jiancheng assay agents (A003–1, A001–3, A064–1-1, A020–1-2, and A005, respectively). The ATP level was analyzed using a Beyotime ATP assay kit (S0026). Routine blood routine tests were performed at the Xinqiao Hospital, Chongqing, China (Fig. 1d).

### Treadmill running experiment for chronic hypoxic rats

**Fig. 2:**
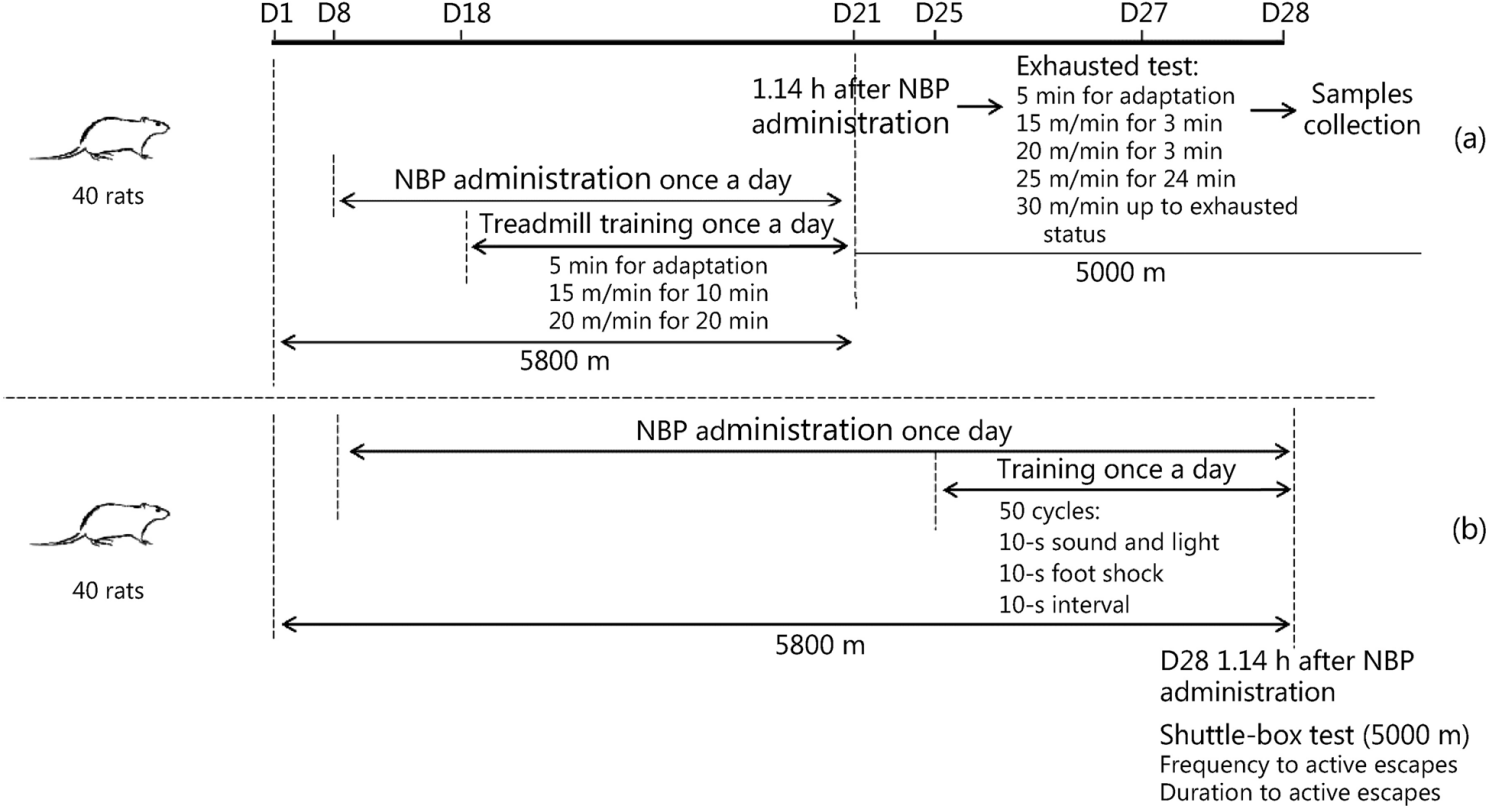
Diagrams depicting experiments conducted under conditions of chronic hypoxia. (**a**) Treadmill running experiment for chronic hypoxic rats; (**b**) shuttle-box experiment for chronic hypoxic rats

This experiment was used to evaluate the effect of NBP on the physical ability of rats under conditions of chronic hypobaric hypoxia. Forty rats were randomly divided to four experimental groups with 10 rats in each group. Rats were placed in a hypobaric hypoxia chamber at 5800 m and intragastrically administered NBP for 13 days from day 8. Rats were exercised on the treadmill as above for 3 days from day 18. On day 21, an exhaustion test was conducted using the treadmill running experiment after NBP administration at 5000 m altitude. At exhausted status, rats were anaesthetized and their arterial blood samples were obtained to analyze MDA, SOD, H_2_O_2_, lactate, and GSH-Px levels and perform routine blood tests. Further, the gastrocnemius muscles were excised for ATP detection (Fig. 2a).

### Shuttle-box experiment for acute hypoxic rats

To investigate the effects of NBP on learning and memory ability under conditions of acute hypoxia, the behavior of rats was evaluated in a shuttle-box (RD1106-SB-R, Shanghai Mobile Datum Information Technology Company, Shanghai, China). Forty rats were randomly divided to four experimental groups with 10 rats in each group. Rats were placed in a hypobaric hypoxia chamber at 5800 m and administered NBP via the intragastric route for 6 days. On day 4, rats were subjected to an exercise program. Each rat underwent 50 trials daily after a 5-min adaptation period. Rats were exposed to a 10-s sound and light signal followed by a 10-s foot shock (2.2 mA) and a 10-s interval in each trial. If the rat moved to the other chamber with the onset of sound and light, the behavior was counted as an active escape and the sound and light signal was turned off with no exposure to foot shock. The time from the start of an active escape to the expected foot shock was regarded as the duration of the active escape. If the rat did not change chambers during a trial, the behavior was counted as an error and the rat received a 10-s foot shock. In each series of 50 trials, the frequency and duration of active escapes were used as the measure of learning and memory [19]. On day 7, the frequency and duration of active escapes of rats were recorded (50 trials) in the shuttle-box experiment 1.14 h after NBP administration in the 5000 m hypobaric hypoxia chamber. Rats were then anaesthetized and their arterial blood was collected to measure MDA, SOD, H_2_O_2_, and GSH-Px levels (Fig. 1e).

### Shuttle-box experiment for chronic hypoxic rats

This experiment was used to evaluate the effect of NBP on learning and memory ability of rats under chronic hypobaric hypoxia. Forty rats were randomly divided to four experimental groups with 10 rats in each group. Rats were placed in a hypobaric hypoxia chamber at 5800 m and administered NBP via the intragastric route for 20 days from day 8. From day 25, rats were subjected to the exercise program described in section 2.7 for 3 days. On day 28, the frequency and duration of active escapes of rats were recorded (50 trials) using the shuttle-box experiment 1.14 h after NBP administration in the 5000 m hypobaric hypoxia chamber. At the end of the experiment, rats were anaesthetized and arterial blood was collected for the analysis of MDA, SOD, H_2_O_2_, and GSH-Px levels (Fig. 2b).

### Statistical analysis

Statistical analysis was carried out using the SPSS 19.0 software. For the survival analysis, a Kaplan-Meier curve was generated using the log-rank test. Other experiments were analyzed using One-way ANOVA test. Data represented mean values of at least three independent experiments ± SD (standard deviation). A value of *P* < 0.05 was considered statistically significant.

## Results

### NBP improved the hypoxia tolerance of rats and mice

**Fig. 3:**
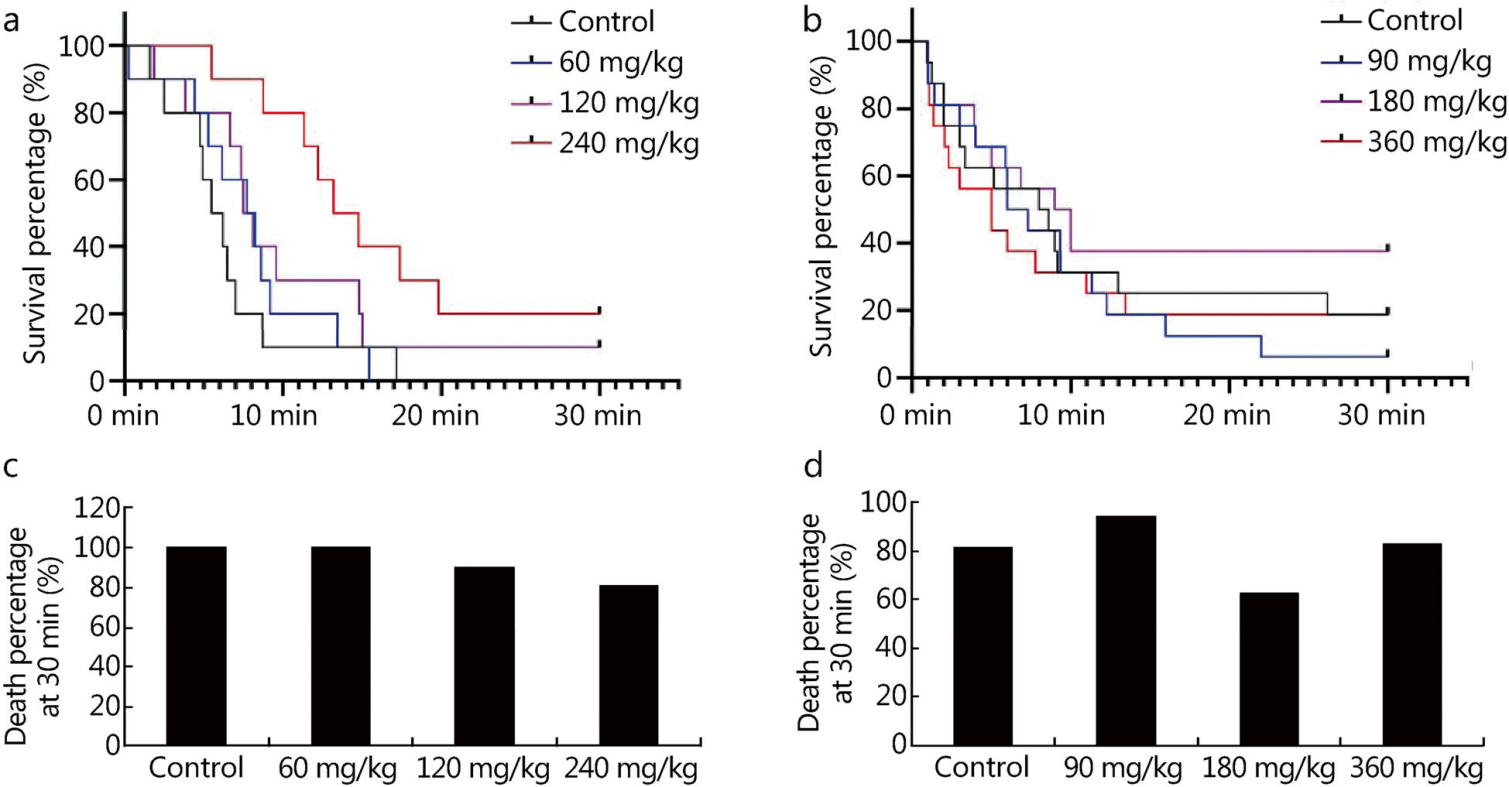
Survival analysis of rats and mice with administration of NBP at 10,000 m exposure. The survival curve of rats (**a**) and mice (**b**) at 10,000 m exposure after NBP administration for 7 days with 30 min as the stop time. Death percentages of rats (**c**) and mice (**d**) after 30 min exposure at 10,000 m

We firstly studied the effect of NBP on survival time at 10,000 m with 30 min as the stop time. The survival curves of the experiments showed that administration of 120 and 240 mg/kg NBP significantly improved the survival times of rats. Compared with the control groups (rats 100%, mice 81.3%), the death percentages at 30 min for the 120 (90.0%) and 240 mg/kg group (80.0%) for rats and the 180 mg/kg group (62.5%) for mice had declined (Fig. 3). Moreover, the standard tolerance time of mice under conditions of closed hypoxia was also markedly extended by 360 mg/kg NBP administration for 5 days (control group, 12.18 ± 2.00 min/(100 ml·g); 360 mg/kg group, 13.94 ± 1.54 min/(100 ml·g); *P* = 0.048; Table S1). These results indicated that NBP could improve the hypoxia tolerance of rats and mice.

### NBP improved physical abilities under conditions of acute and chronic hypoxia

**Fig. 4:**
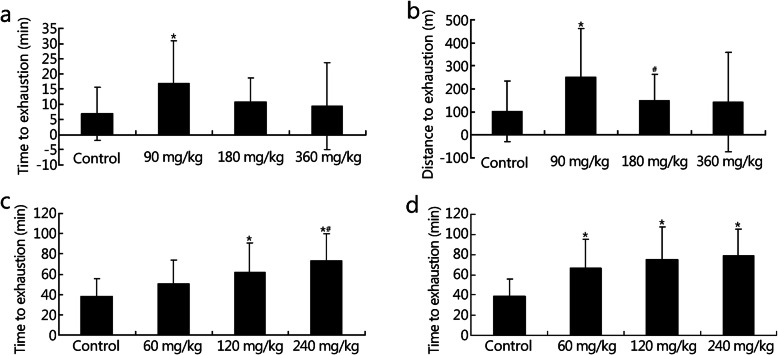
Effects of NBP on the time and distance to motor exhaustion of mice and rats under conditions of acute and chronic hypoxia. Time (**a**) and distance (**b**) to exhaustion of mice in the forced exercise wheel-track treadmill experiment at 5800 m for 3 days. Time to exhaustion of rats in the treadmill running experiment at 5800 m for 3 days (**c**) and 20 days (**d**). ^*^P < 0.05 compared with control group; ^#^*P* < 0.05 compared with 60 mg/kg group

Under acute hypoxic conditions, 90 mg/kg NBP significantly improved the time to exhaustion (control group, 6.79 ± 8.72 min; 90 mg/kg group, 16.79 ± 14.00 min; *P* = 0.012) and distance to exhaustion (control group, 101.62 ± 130.78 m; 90 mg/kg group, 251.49 ± 210.07 m; *P* = 0.011) of mice (Fig. 4a-b) and NBP treatment at 120 and 240 mg/kg concentrations significantly increased the time to exhaustion of rats (control group, 38.28 ± 17.85 min; 120 mg/kg group, 61.80 ± 19.30 min; *P* = 0.024; 240 mg/kg group, 73.26 ± 26.89 min; *P* = 0.001; Fig. 4c). Moreover, NBP at 60, 120, and 240 mg/kg concentrations significantly increased the time to exhaustion of rats under conditions of chronic hypoxia (control group, 38.59 ± 16.83 min; 60 mg/kg group, 66.83 ± 28.10 min; *P* = 0.014; 120 mg/kg group, 75.00 ± 32.34 min; *P* = 0.002; 240 mg/kg group, 79.10 ± 26.33 min; *P* = 0.001; Fig. 4d). These results suggest that NBP may improve the physical ability of rodents under conditions of acute and chronic hypoxia.

**Fig. 5:**
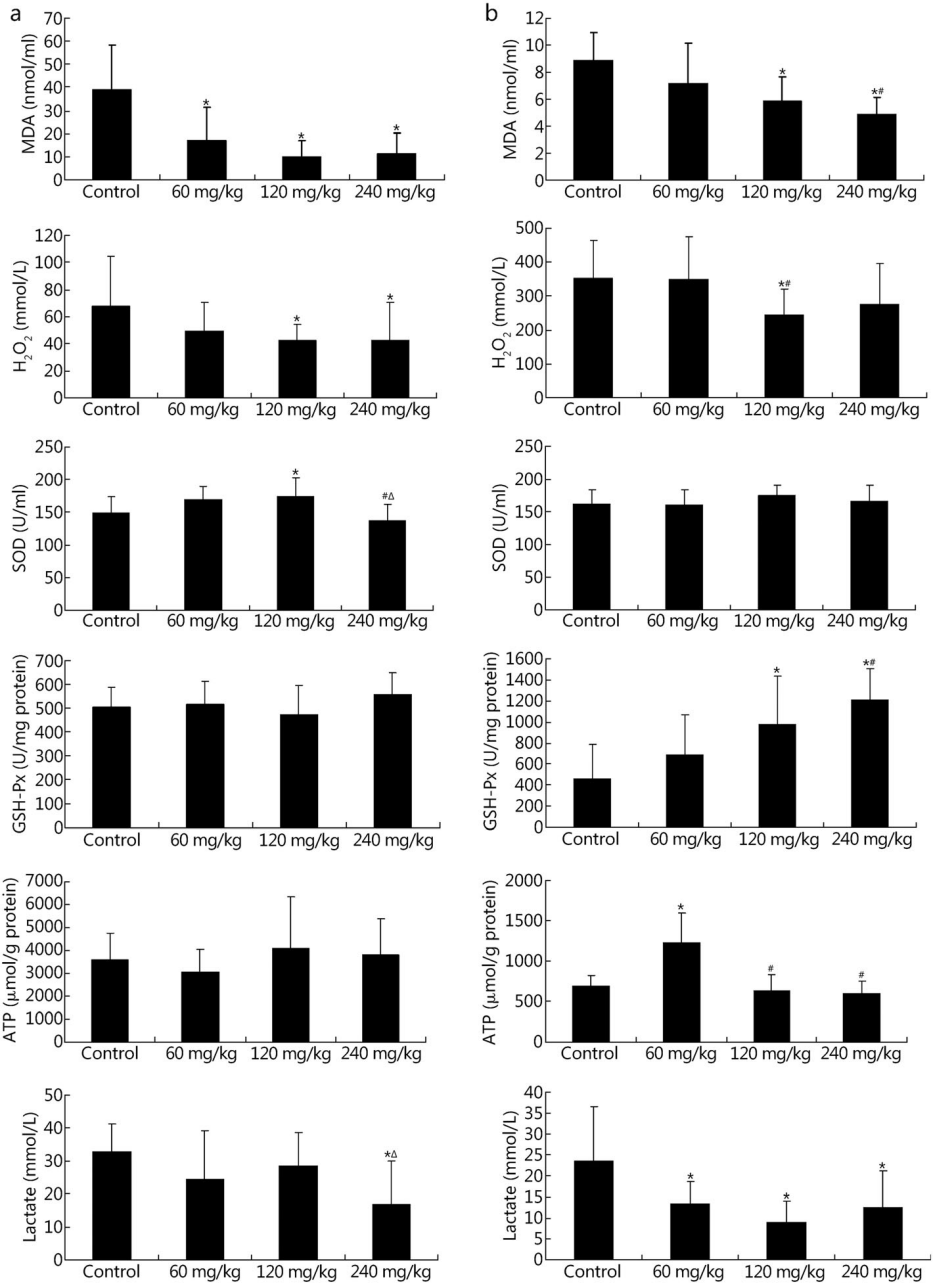
Effects of NBP on the energy metabolism and oxidative stress of exhausted rats under conditions of acute and chronic hypoxia. MDA, H_2_O_2_, SOD, GSH-Px, and lactate levels in the serum and ATP levels in the gastrocnemius muscle of acute (**a**) and chronic (**b**) hypoxic rats were detected. ^*^P < 0.05 compared with control group; ^#^*P* < 0.05 compared with 60 mg/kg group; ^∆^*P* < 0.05 compared with 120 mg/kg group

To clarify the mechanism underlying the NBP-mediated acceleration in physical activity under acute and chronic hypoxic conditions, we evaluated the levels of MDA, H_2_O_2_, SOD, GSH-Px, and lactate in the blood and ATP in the gastrocnemius muscle of rats. The levels of MDA (control group, 38.83 ± 19.59 nmol/ml; 60 mg/kg group, 16.89 ± 14.48 nmol/ml; *P* < 0.001; 120 mg/kg group, 9.98 ± 6.95 nmol/ml; *P* < 0.001; 240 mg/kg group, 11.11 ± 9.17 nmol/ml; *P* < 0.001) and H_2_O_2_ (control group, 67.84 ± 36.43 mmol/L; 120 mg/kg group, 42.44 ± 12.26 mmol/L; *P* = 0.031; 240 mg/kg group, 42.36 ± 28.25 mmol/L; *P* = 0.027) decreased but that of SOD (control group, 148.47 ± 24.80 U/ml; 120 mg/kg group, 173.78 ± 29.33 U/ml; *P* = 0.020) increased with no obvious change of GSH-Px levels under conditions of acute hypoxia following treatment with various doses of NBP (Fig. 5a). Under conditions of chronic hypoxia, levels of MDA (control group, 8.84 ± 2.06 nmol/ml; 120 mg/kg group, 5.88 ± 1.75 nmol/ml; *P* = 0.002; 240 mg/kg group, 4.86 ± 1.27 nmol/ml; *P* < 0.001) and H_2_O_2_ (control group, 351.13 ± 111.57 mmol/L; 120 mg/kg group, 245.28 ± 76.66 mmol/L; *P* = 0.037) decreased, but GSH-Px expression was upregulated (control group, 457.01 ± 326.03 U/mg protein; 120 mg/kg group, 976.35 ± 462.23 U/mg protein; *P* = 0.014; 240 mg/kg group, 1210.58 ± 294.57 U/mg protein; *P* = 0.002) with no obvious change of SOD levels (Fig. 5b). Thus, NBP may exert opposite effects by increasing the anti-oxidant capacity of the rats and decreasing the oxidant capacity. The lactate levels significantly decreased under conditions of acute hypoxia (control group, 32.79 ± 8.63 mmol/L; 240 mg/kg group, 16.83 ± 13.14 mmol/L; *P* = 0.002) and chronic hypoxia (control group, 23.50 ± 13.03 mmol/L; 60 mg/kg group, 13.38 ± 5.15 mmol/L; *P* = 0.010; 120 mg/kg group, 8.90 ± 5.01 mmol/L; *P* < 0.001; 240 mg/kg group, 12.48 ± 8.65 mmol/L; *P* = 0.008), whereas the levels of ATP (control group, 686.55 ± 129.51 μmol/g protein; 60 mg/kg group, 1228.51 ± 364.34 μmol/g protein; *P* < 0.001) significantly increased under conditions of chronic hypoxia but not acute hypoxia (Fig. 5a-b). These observations suggest NBP promoted ATP production via oxidative phosphorylation instead of glycolysis. NBP at a dose of 240 mg/kg significantly decreased the red blood cell (*P* = 0.007), hemoglobin (*P* = 0.010), and platelet counts (*P* = 0.002) as well as the hematocrit level (*P* = 0.025) under conditions of acute hypoxia (Table S2). However, NBP significantly increased the white blood cell count at a dose of 60 mg/kg (*P* = 0.044) under conditions of chronic hypoxia (Table S3).

### NBP improved learning and memory abilities under conditions of acute and chronic hypoxia

**Fig. 6:**
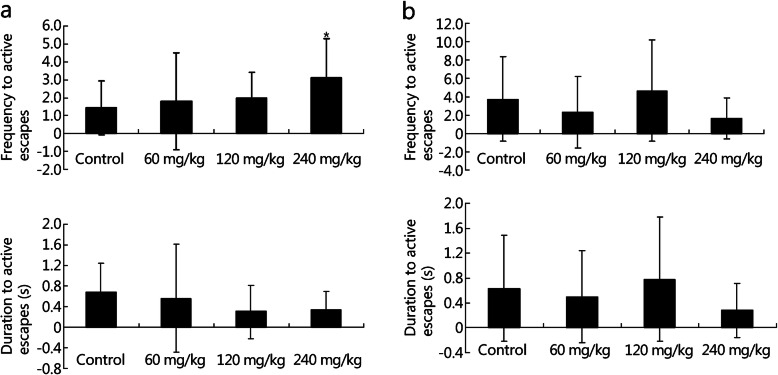
Effects of NBP on rats under conditions of acute and chronic hypoxia in the shuttle-box experiment. Frequency of active escapes and duration of active escapes for rats at 5800 m for 6 days (**a**) and 27 days (**b**) in the shuttle-box experiments. ^*^P < 0.05 compared with control group

**Fig. 7:**
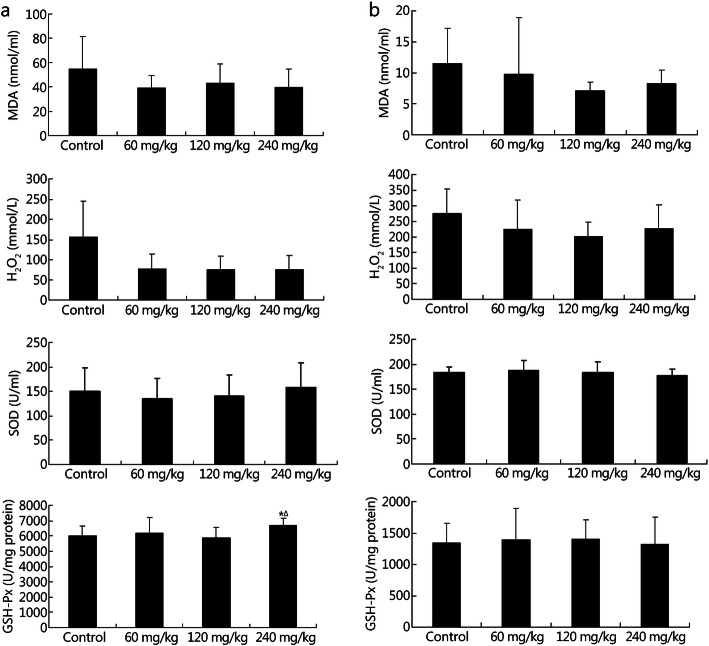
Effects of NBP on the oxidative stress of rats under conditions of acute and chronic hypoxia after the shuttle-box experiment. Levels of MDA, H_2_O_2_, SOD, and GSH-Px in the serum of acute (**a**) and chronic (**b**) hypoxia rats were detected. ^*^P < 0.05 compared with control group; ^∆^*P* < 0.05 compared with 120 mg/kg group

The cognitive functions of the rats were evaluated using a shuttle-box experiment. Under conditions of acute hypoxia, NBP at a dose of 240 mg/kg significantly increased the frequency of active escapes (control group, 1.44 ± 1.50; 240 mg/kg group, 3.13 ± 2.17; *P* = 0.046) with no effect on the duration of active escapes (Fig. 6a). And NBP showed no effects on the frequency and duration of active escapes under conditions of chronic hypoxia (Fig. 6b). The beneficial effects of NBP on learning and memory function were not as consistent as those on physical activity. Furthermore, MDA, H_2_O_2_, SOD, and GSH-Px levels were analyzed in rodent blood samples. The expression of GSH-Px (control group, 5999.26 ± 659.23 U/mg protein; 240 mg/kg group, 6704.55 ± 451.45 U/mg protein; *P* = 0.045) was upregulated with no effects on MDA, H_2_O_2_ and SOD levels (Fig. 7a) under conditions of acute hypoxia. Under conditions of chronic hypoxia, the levels of MDA H_2_O_2_, SOD and GSH-Px levels showed no obvious change (Fig. 7b). Thus, NBP increased the anti-oxidant capacity of the rats during cognitive tests.

## Discussion

High altitude is characterized by a decline in air pressure and partial oxygen pressure. Physical and cognitive functions may be severely affected following exposure to high altitude. Nowadays, the number of people who remain at high altitude for prolonged periods is increasing although it is acknowledged that their work performance and daily life are greatly affected by high altitude. Therefore, there is an urgent need to develop methods that alleviate the physical and cognitive impairments that occur at high altitude in a hypobaric hypoxic environment.

Several studies have demonstrated that NBP ameliorates ischemic injury and promotes neuroplasticity and recovery from injury. The therapeutic mechanisms underlying these effects are closely associated with various processes, including anti-oxidant and anti-inflammation activities, angiogenesis, anti-thrombosis, neurogenesis, and metabolic reprogramming. According to our results above, the effects of NBP on hypobaric hypoxia were mainly related to its anti-oxidant properties and metabolic reprogramming. The failure of human physiological responses to hypobaric hypoxia may result in increased inflammation, oxidative stress, and metabolic adjustment. Our study found that exposure to high altitude resulted in upregulation in the expression of inflammatory cytokines and downregulation in the expression of anti-inflammatory cytokines [7]. Moreover, hypobaric hypoxia induced oxidative damage and decreased antioxidative functions [20]. Metabolic modulation, may cause glycolytic capacity to be promoted and oxidative metabolism to be suppressed in response to hypoxia [21, 22]. A recent study demonstrated the relationship between oxidative stress and accelerated cognitive decline in chronic mountain sickness [23], suggesting that the molecular changes induced by hypobaric hypoxia may lead to behavioral abnormality. Given the effect of NBP on rodents with hypobaric hypoxia, we conclude that NBP reverses the alterations in hypoxia-induced oxidative stress and metabolism, thereby possibly reversing physical and learning and memory changes. Whether NBP could improve the effect of hypoxia on inflammation or not warrants further study.

The effects and mechanisms of acute and chronic hypoxia on humans and animals are remarkably different [24–27], and treatments for reducing damage or diseases due to acute and chronic hypoxia also show great discrepancies. However, in this work, there were no obvious differences in the effects of NBP on acute and chronic hypoxia. There could be a common mechanism underlying acute and chronic hypoxia, so that some therapies and agents, such as NBP, could improve certain abilities under conditions of both acute and chronic hypoxia. Moreover, the effects of hypoxia on the brain display region-specific characteristics [28, 29]. We studied the effects of NBP on learning and memory functions [30, 31], which commonly decline under these conditions, to evaluate the effects of this agent on cognitive ability. The effects of NBP on other cognitive functions under conditions of hypobaric hypoxia remain to be investigated.

While NBP played an important role in improving the physical and learning and memory functions of rodents under hypobaric hypoxia, it would be interesting to assess the effects of NBP on diverse cognitive functions at high-altitude (and after exposure). However, the dosage administered in this study was relatively higher than those employed in other studies. Therefore, it is imperative to refine the NBP treatment and develop new therapeutics for reducing hypobaric hypoxia-induced physical and cognitive decline.

## Conclusion

In summary, we demonstrated the NBP-mediated improvement in physical and learning and memory abilities of rodents under conditions of acute and chronic hypobaric hypoxia, as evidence from the elevation in anti-oxidative functions and promotion of oxidative phosphorylation rather than glycolysis.

## Supplementary Information

**Additional file 1: Figure S1.** Structure of DL-3-n-butylphthalide.

**Additional file 2: Table S1.** Standard tolerance times (min/(100 ml·g)) of mice under conditions of closed hypoxia and administration of NBP and closed hypoxia at 3, 5, 7 days (mean ± SD). ^*^P < 0.05 compared with control group.

**Additional file 3: Table S2.** Effects of NBP on routine blood tests of exhausted rats under conditions of acute hypoxia (mean ± SD). WBC. White blood cell; HCT. Hematocrit; RBC. Red blood cell; MCH. Mean corpuscular hemoglobin; HGB. Hemoglobin; MCHC. Mean corpuscular hemoglobin concentration; MCV. Mean corpuscular volume; PLT. Platelet count. ^*^P < 0.05 compared with control group; ^#^*P* < 0.05 compared with 60 mg/kg group; ^∆^*P* < 0.05 compared with 120 mg/kg group.

**Additional file 4: Table S3.** Effects of NBP on routine blood tests of exhausted rats under conditions of chronic hypoxia (mean ± SD). WBC. White blood cell; HCT. Hematocrit; RBC. Red blood cell; MCH. Mean corpuscular hemoglobin; HGB. Hemoglobin; MCHC. Mean corpuscular hemoglobin concentration; MCV. Mean corpuscular volume; PLT. Platelet count. ^*^P < 0.05 compared with control group; ^#^*P* < 0.05 compared with 60 mg/kg group.

## Data Availability

Not applicable.

## Abbreviations

BW: Body weight
GSH-Px: Glutathione peroxidase
H_2_O_2_: Hydrogen peroxide
MDA: Malonaldehyde
NBP: DL-3-n-butylphthalide
SD: Sprague-Dawley
SOD: Superoxide dismutase
ST: Survival time
STT: Standard hypoxia tolerance time
V: Volume
