# Supplementary figures and images for: DL-3-n-butylphthalide improved physical and learning and memory performance of rodents exposed to acute and chronic hypobaric hypoxia
Source: Mil Med Res. 2021 Mar 25;8:23. doi: 10.1186/s40779-021-00314-7 (PMC7993509; doi:10.1186/s40779-021-00314-7)

**
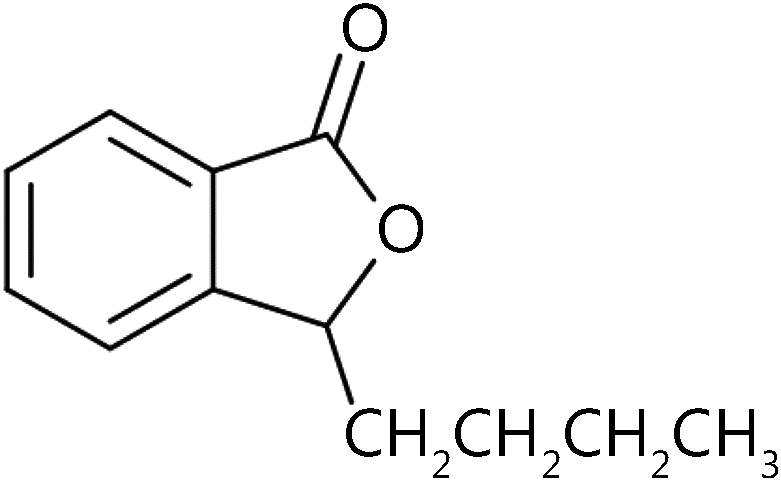
**

**Fig. S1** Structure of DL-3-n-butylphthalide.

Supplement: Supplementary file 1 — Additional file 1: Figure S1. Structure of DL-3-n-butylphthalide. [file 40779_2021_314_MOESM1_ESM.docx]
