# Supplementary material for: DL-3-n-butylphthalide improved physical and learning and memory performance of rodents exposed to acute and chronic hypobaric hypoxia
Source: Mil Med Res. 2021 Mar 25;8:23. doi: 10.1186/s40779-021-00314-7 (PMC7993509; doi:10.1186/s40779-021-00314-7)
Supplement: Supplementary file 2 — Additional file 2: Table S1. Standard tolerance times (min/(100 ml·g)) of mice under conditions of closed hypoxia and administration of NBP and closed hypoxia at 3, 5, 7 days (mean ± SD). *P < 0.05 compared with control group. [file 40779_2021_314_MOESM2_ESM.docx]

**Table S1** Standard tolerance times (min/(100ml·g)) of mice under conditions of closed hypoxia and administration of NBP and closed hypoxia at 3, 5, 7 days (mean± SD)

| Group | Day 3 | Day 5 | Day 7 |
| --- | --- | --- | --- |
| Control | 13.49±2.97 | 12.18±2.00 | 10.54±1.81 |
| 90 mg/kg | 11.92±3.76 | 13.32±2.52 | 10.21±1.24 |
| 180 mg/kg | 13.18±1.70 | 13.91±2.14 | 10.63±1.69 |
| 360 mg/kg | 14.35±1.75 | 13.94±1.54^*^ | 11.47±1.81 |

^*^*P*<0.05 compared with control group.
