# Supplementary material for: DL-3-n-butylphthalide improved physical and learning and memory performance of rodents exposed to acute and chronic hypobaric hypoxia
Source: Mil Med Res. 2021 Mar 25;8:23. doi: 10.1186/s40779-021-00314-7 (PMC7993509; doi:10.1186/s40779-021-00314-7)
Supplement: Supplementary file 3 — Additional file 3: Table S2. Effects of NBP on routine blood tests of exhausted rats under conditions of acute hypoxia (mean ± SD). WBC. White blood cell; HCT. Hematocrit; RBC. Red blood cell; MCH. Mean corpuscular hemoglobin; HGB. Hemoglobin; MCHC. Mean corpuscular hemoglobin concentration; MCV. Mean corpuscular volume; PLT. Platelet count. *P < 0.05 compared with control group; #P < 0.05 compared with 60 mg/kg group; ∆P < 0.05 compared with 120 mg/kg group. [file 40779_2021_314_MOESM3_ESM.docx]

**Table S2** Effects of NBP on routine blood tests of exhausted rats under conditions of acute hypoxia (mean± SD)

| Group | WBC(×10^9^/L) | HCT(mm/h) | RBC(×10^12^/L) | MCH(pg) | HGB(g/L) | MCHC(g/L) | MCV(fl) | PLT(×10^12^/L) |
| --- | --- | --- | --- | --- | --- | --- | --- | --- |
| Control | 14.7±5.2 | 60.5±3.3 | 8.2±0.6 | 21.5±0.7 | 176±10 | 293±7 | 73.4±4.3 | 1.34±0.17 |
| 60 mg/kg | 13.9±3.6 | 61.2±5.3 | 8.1±0.6 | 21.6±0.8 | 174±9 | 285±13 | 75.8±4.8 | 1.31±0.25 |
| 120 mg/kg | 14.9±4.9 | 60.3±7.8 | 8.2±0.9 | 20.1±0.7 | 171±15 | 285±15 | 73.4±3.5 | 1.33±0.16 |
| 240 mg/kg | 16.3±9.7 | 54.1±5.6^*#∆^ | 7.3±0.5^*#∆^ | 22.0±0.6 | 161±7^*^ | 300±22 | 73.9±5.8 | 1.13±0.13^*#∆^ |

WBC. White blood cell; HCT. Hematocrit; RBC. Red blood cell; MCH. Mean corpuscular hemoglobin; HGB. Hemoglobin; MCHC. Mean corpuscular hemoglobin concentration; MCV. Mean corpuscular volume; PLT. Platelet count. ^*^*P*<0.05 compared with control group; ^#^*P*<0.05 compared with 60 mg/kg group. ^*^*P*<0.05 compared with control group; ^#^*P*<0.05 compared with 60 mg/kg group; ^∆^*P*<0.05 compared with 120 mg/kg group.
