# Supplementary material for: DL-3-n-butylphthalide improved physical and learning and memory performance of rodents exposed to acute and chronic hypobaric hypoxia
Source: Mil Med Res. 2021 Mar 25;8:23. doi: 10.1186/s40779-021-00314-7 (PMC7993509; doi:10.1186/s40779-021-00314-7)
Supplement: Supplementary file 4 — Additional file 4: Table S3. Effects of NBP on routine blood tests of exhausted rats under conditions of chronic hypoxia (mean ± SD). WBC. White blood cell; HCT. Hematocrit; RBC. Red blood cell; MCH. Mean corpuscular hemoglobin; HGB. Hemoglobin; MCHC. Mean corpuscular hemoglobin concentration; MCV. Mean corpuscular volume; PLT. Platelet count. *P < 0.05 compared with control group; #P < 0.05 compared with 60 mg/kg group. [file 40779_2021_314_MOESM4_ESM.docx]

**Table S3** Effects of NBP on routine blood tests of exhausted rats under conditions of chronic hypoxia (mean± SD)

| Group | WBC(×10^9^/L) | HCT(mm/h) | RBC(×10^12^/L) | MCH(pg) | HGB(g/L) | MCHC(g/L) | MCV(fl) | PLT(×10^12^/L) |
| --- | --- | --- | --- | --- | --- | --- | --- | --- |
| Control | 6.8±2.9 | 70.7±6.0 | 10.5±0.8 | 22.2±2.1 | 233±18 | 330±13 | 67.4±8.1 | 0.58±0.19 |
| 60 mg/kg | 9.5±2.7^*^ | 73.0±4.8 | 10.5±1.0 | 23.0±1.8 | 238±10 | 327±17 | 70.4±9.2 | 0.50±0.13 |
| 120 mg/kg | 6.7±3.7^#^ | 73.4±3.9 | 10.9±1.0 | 22.4±1.7 | 243±12 | 332±14 | 67.8±7.1 | 0.44±0.07 |
| 240 mg/kg | 4.9±1.6^#^ | 71.6±3.4 | 10.7±0.5 | 22.3±1.0 | 238±11 | 332±10 | 67.2±4.2 | 0.50±0.12 |

WBC. White blood cell; HCT. Hematocrit; RBC. Red blood cell; MCH. Mean corpuscular hemoglobin; HGB. Hemoglobin; MCHC. Mean corpuscular hemoglobin concentration; MCV. Mean corpuscular volume; PLT. Platelet count. ^*^*P*<0.05 compared with control group; ^#^*P*<0.05 compared with 60 mg/kg group. ^*^*P*<0.05 compared with control group; ^#^*P*<0.05 compared with 60 mg/kg group.
